# Recent Advances in Hemophilia Gene Therapy

**Published:** 2019-01

**Authors:** Morteza Karimipoor

**Affiliations:** Department of Molecular Medicine, Biotechnology Research Center, Pasteur Institute of Iran, Tehran, Iran

Heterogeneous loss of function mutations at *F8* and *F9* genes causes X-linked recessive bleeding disorders, hemophilia A (HA) and hemophilia B (HB), respectively. HA is clinically indistinguishable from HB and accounts for more than 80% of hemophilia cases; the former affects 1/5000 and the latter 1/25000 male births worldwide. In Iran, it is estimated that around 4300 HA and 900 HB patients are under care. The standard care for hemophilia patients is lifelong on demand or prophylactic factor replacement therapy. However, the complications such as arthropathies in severe form of the disease and viral infections are not uncommon. The cost of prophylactic treatment is high and not affordable in developing countries. The annual cost of hemophilia treatment in Iran is not predictable, but it is estimated to be around 50,000-60,000 US dollars/patient.

In last two decades, preclinical and clinical studies have been performed to evaluate the safety and efficacy of the adeno-associated virus (AAV) particles containing *F8* and *F9* genes, as a novel modality of hemophilia treatment. AAV is a single-stranded DNA virus (4.7 kb) and mainly is not associated with any manifestations. The AAV genome contains *rep* and *cap* genes flanked by two inverted terminal repeats at both ends. These two genes in AAV vectors, used for gene therapy purposes, are replaced by the gene of interest and inserted into packaging vector in *trans*. In addition, helper plasmid is necessary for the production of AAV vector particles. The advantages of the AAV vectors include the lack of immunogenicity, transduction of dividing and non-dividing cells, tropism of different serotypes of the virus to different organs in the body, and the lack of integration of vector DNA into human genome. However, the limited capacity of the vector and pre-existing neutralizing antibodies (NAb) in population (20-80% of the population regarding to the serotype) are the downsides of the AAV vectors.

Hemophilia is an ideal target for gene therapy using AAV vectors. Increasing the factor activity from 1% to 5% changes the disease from severe form of the disease to mild form. The molecular basis of gene regulation of *F8* and *F9* is not complicated, and preclinical studies using AAV vectors have been promising, especially when employing serotypes five and eight of the vector that has tropism to liver. The goal of gene therapy in hemophilia is to reach the factor activity to more than 12%, as the previous data has been shown the prevention of arthropathy at this level.

The first clinical study of gene therapy using AAV vector for HB utilized AAV2 serotype containing F9 cDNA by multiple intramuscular injections in a phase I/II study. Although the trial was not associated with adverse effects, the plasma FIX level more than 1% was not obtained in seven patients enrolled in the study. In the second trial using AAV vector, AAV2 particles containing FIX expression cassette was infused into hepatic artery over three different doses of vector genome per kilogram (vg/kg). In two patients received high dose of the vector, the plasma FIX levels reached 4% and 10% following four weeks of vector administration and then declined to the baseline level. The decline of FIX activity to the baseline level was related with the increased hepatic transaminases in one patient. The transaminitis was self-limited, and it has been hypothesized that cytotoxic T-cells react against transduced hepatocytes that present capsid peptides associated with MHC class I molecules on the surface.

The first successful HB gene therapy trial using AAV was published by Nathwani and colleagues in 2011. To get rid of immune system against AAV2 capsid, they used AAV2/8 pseudotype vector containing codon optimized F9 cDNA downstream of a liver-specific promoter. Another reason of using capsid serotype 8 was the high tropism of AAV8 to liver. They used self-complementary AAV to increase the efficiency of expression. The low, intermediate, and high dose of vector was individually infused in the peripheral vein of six severe HB patients. The FIX expression was 2%-11% of normal levels, and four patients discontinued prophylactic treatment. Two patients receiving the high dose of the vector showed high levels of hepatic aminotransferase responded to corticosteroids. Longer follow-up to six years showed stable expression of FIX in the patients. In 2014, four further subjects were infused with the high dose of the same vector, and three of them discontinued prophylaxis (FIX levels 5% to 8%), and in one patient the FIX level declined to 2%. Further purification of the vector and not using empty capsid are planned to extend the study in the future. The results demonstrated the immune response to capsid is related to AAV vector dose, and attempt should be directed to the use of the low doses of the vector.

Recently, the data of a gene therapy trial using highly active variant of FIX has been reported. FIX-R338L (Padua variant) is a natural highly specific active variant of FIX with more than eightfold thrombotic activity. Hence, it could be used in the low dose of vector, lowering the risk of immune response against AAV capsid. In a previous study, AAV vector consisting of a bioengineered capsid, liver-specific promoter, and FIX-Padua was infused to peripheral vein of 10 HB patients. The follow-up of the patients (28-78 weeks) showed the sustained activity of FIX between 14% to 81% of normal levels. The data also confirmed the preclinical studies that the Padua variant does not increase the risk of inhibitor formation. In another study, the AAV5 vector was applied in two doses (5 × 10^12^ or 2 × 10^13^ genome copies of AMT-060/kilogram) for the treatment of 10 patients with HB. The vector was manufactured in insect cells using a baculovirus expression system. The authors concluded that the vector was safe in terms of capsid specific T-cell response, and also sufficient FIX activity in the subjects was obtained.

Besides aforementioned trials, there are a few trials using AAV for the treatment of HB, which the data have not been published in peer-reviewed journals. AAV vectors have also been used for HA gene therapy. The coding size of *F8* gene (about 7 kb) is above the limitation size of AAV packaging (4.7 kb), and the expression of FVIII is not efficient. The preclinical studies have revealed that the B-domain-deleted FVIII (BDD-FVIII) improves the mRNA level 17folds and secretion efficiency more than 30%; B-domain is not required for co-factor activity of FVIII in tenase complex for clot formation. An investigation has reported the safety and efficacy of AAV5 containing BDD-hFVIII-SQ under the control of liver-specific hybrid promoter in nine HA patients in three-dose-cohort. In high dose (6 × 10^13^ vg/kg) at week 52, the median activity of FVIII was 77 IU/dl. Increase in FVIII activity was dose-dependent and in the high-dose cohort, six out of seven subjects had normal physiologic levels of FVIII activity. One interesting point of the study is that FVIII expression takes longer time to reach the steady state compared to FIX and other serotypes.

In an attempt to treat HA, George *et al*. used recombinant AAV vector composed of bio-engineered capsid (SPK-8011), as a single intravenous infusion with starting dose of 5 × 10^11^ vg/kg. The preliminary results of three patients included in the study showed they have FVIII activity of 12% and discontinued the prophylaxis. No serious adverse event including T-cell-mediated cytotoxicity against hepatocytes was observed.

Overall, the data of gene therapy trials of hemophilia in last six years and mainly in last two years are promising, and ongoing trials will shed more light on the safety and efficacy profile of the vectors. The cost of good manufacturing practice grade of AAV vector production and purification in mammalian and insect cells and affordability of treatment are main concerns, especially for the patients at least in developed or developing countries. Pre-existing NAbs against natural serotypes of vector in the population is an obstacle for the enrolment of the patients in the trial studies. Humoral and cellular immunity against administered vectors and transgene in the patients, the persistence of gene expression, and probable need to re-administration of the vector, the tropism of the vectors to other organs and tissues, germ line transmission of the vector, the effect of integrated vectors on genome of the patients, and the risk of mutagenesis are current or future concerns regarding the AAV-based gene therapy. Resolving these concerns may lead to the approval of gene therapy of hemophilia using AAV vectors by FDA in the near future.

**More details in:**

1. ***Gene therapy for hemophilia***. AC Nathwani et al. 2017. Hematol Oncol Clin North Am; Vol. 31, 853-868.
2. ***Emerging issues in AAV-mediated in vivo gene therapy***. P Colella P, et al. 2017. Mol Ther Methods Clin Dev; Vol. 8, 87-104.
3. ***Gene therapy for hemophilia: what does the future hold*?** BS Doshi, et al. 2018. Ther Adv Hematol; Vol. 9, 273-293.
4. ***Advances in gene therapy for hemophilia: basis, current status, and future perspectives***. T Ohmori. 2018. Int J Hematol; doi: 10.1007/s12185-018-2513-4.
5. ***Gene therapy comes of age***. CE Dunbar CE, et al. 2018. Science; Vol. 359, pii: eaan4672.

